# Bacterial cellulose films with ZnO nanoparticles and propolis extracts: Synergistic antimicrobial effect

**DOI:** 10.1038/s41598-019-54118-w

**Published:** 2019-11-27

**Authors:** Alexandra Mocanu, Gabriela Isopencu, Cristina Busuioc, Oana-Maria Popa, Paul Dietrich, Liana Socaciu-Siebert

**Affiliations:** 1 University POLITEHNICA of Bucharest, Faculty of Applied Chemistry and Materials Science, Gh. Polizu Street 1-7, postal code 011061, Bucharest, Romania; 2 Research Center for Instrumental Analysis – SCIENT, Petre Ispirescu Street 1, Tâncăbeşti, postal code 077167, Ilfov, Romania; 3 SPECS Surface Nano Analysis GmbH, Voltastrasse 5, 13355 Berlin, Germany

**Keywords:** Biomaterials, Biomaterials

## Abstract

This study aimed to obtain possible materials for future antimicrobial food packaging applications based on biodegradable bacterial cellulose (BC). BC is a fermentation product obtained by *Gluconacetobacter xylinum* using food or agricultural wastes as substrate. In this work we investigated the synergistic effect of zinc oxide nanoparticles (ZnO NPs) and propolis extracts deposited on BC. ZnO NPs were generated in the presence of ultrasounds directly on the surface of BC films. The BC-ZnO composites were further impregnated with ethanolic propolis extracts (EEP) with different concentrations.The composition of raw propolis and EEP were previously determined by gas-chromatography mass-spectrometry (GC-MS), while the antioxidant activity was evaluated by TEAC (Trolox equivalent antioxidant capacity). The analysis methods performed on BC-ZnO composites such as scanning electron microscopy (SEM), thermo-gravimetrically analysis (TGA), and energy-dispersive X-ray spectroscopy (EDX) proved that ZnO NPs were formed and embedded in the whole structure of BC films. The BC-ZnO-propolis films were characterized by SEM and X-ray photon spectroscopy (XPS) in order to investigate the surface modifications. The antimicrobial synergistic effect of the BC-ZnO-propolis films were evaluated against *Escherichia coli*, *Bacillus subtilis*, and *Candida albicans*. The experimental results revealed that BC-ZnO had no influence on Gram-negative and eukaryotic cells.

## Introduction

Microbiological contamination represents a continuous issue in applications that require sterile conditions. The constant adaptation of microorganism to different environmental conditions generated in the last century infection agents resistant to drugs or antibiotics^1–3^. Thus, numerous research studies have been directed towards manufacturing of new drugs on one hand, or materials with strict requests in terms of environment safety on the other.

Nowadays, the design of antibacterial or antimicrobial materials implies several important aspects, such as: (i) the use of cheap raw materials and agricultural or food wastes; (ii) facile synthesis and processing method; (iii) biocompatible properties in the case of medical or food applications; (iv) reduced impact on the environment at the end of the life span; (v) manufacturing of biodegradable products.

Bacterial cellulose (BC) results from the fermentation processes of different biomass residues in the presence of *Gluconacetobacter xylinum* assigned as *Acetobacter xylinum* bacteria^4^. Its remarkable properties such as high retention of water, high mechanical and thermal resistance, and crystalline structure due to the 3D irregular disposal of BC nanofibers^5^ made it suitable for different industrial fields.

Thus, BC and BC composite materials have gained a lot of attention not only in water splitting or waste treatment applications^6,7^, proton conducting membranes^8^, supercapacitors, solar cells and transparent displays^9,10^ but also in the field of wound dressings^11,12^, food packaging^13–16^, tissue engineering^17,18^ and superabsorbent in agriculture applications to improve the pedoclimate conditions for arid soils^19^.

The development of BC-based materials involved also antimicrobial applications, although pristine BC has no antimicrobial properties. For this reason, considerable effort has been made to modify BC in order to increase such characteristics. These methods include grafting of aldehyde groups on the surface^20^, modification with different inorganic nanoparticles that exhibit antimicrobial properties (ZnO, TiO_2_, CuO, Ag nanoparticles, etc.)^21,22^ or immobilization of different peptides^12,23^. Furthermore, recent studies involved the tailoring of various polymer films or commercially food packages with essential oils which are known as natural antimicrobial agents and rated as safe for medical and food applications by European Union^24,25^.

Inorganic material ZnO was involved in numerous commercial applications related to electronic and optical devices, coatings and dyes, cosmetics, drugs, photocatalytic degradation processes of organic dyes for wastewater treatment^26–29^, due to its wide bandgap (3.4 eV), large excitation of binding energy (60 meV), and high thermal stability^30^.

In food engineering applications, ZnO NPs has gained much attention in research studies due to its intrinsic antimicrobial properties and low toxicity to human body^15,31,32^.

It is well known, that the nanocomposites of BC with ZnO NPs shows antibacterial activity. Until now several techniques were employed for enhancing the antibacterial properties of BC films such as: (**a**) immersion of BC films into aqueous dispersion of commercial ZnO NPs; (**b**) generation of ZnO NPs by hydrothermal or sol-gel processes using zinc(II) acetate or zinc(II) nitrate as precursor in the presence of BC films^33–35^; (**c**) ZnO NPs deposit on BC dry and wet films by matrix-assisted-pulsed-light-evaporation (MAPLE)^36^.

Also, the extracts of essential oils such as curcumin, eugenol or thymol were used to induce antimicrobial properties of food packages against bacterial strains and extend the life time of food products^25,37–39^.

It is well known that propolis is a natural resinous bee product with more than 300 components produced by bees to seal and protect their hive against pathogenic agents^40,41^. Based on these characteristics, propolis extracts were used until now to study their biological effect in terms of antimicrobial materials^42^ or biomedical implants^43^.

Thus, the novelty of this work was to use biodegradale BC films modified with ZnO NPs and EEP using propolis from *Dolj County*, *Romania* for possible applications as food packaging with antimicrobial properties. The motivation for designing such materials was based on the simplicity of the coating/deposition methods compared to other studies^44^ and on the use of biodegradable organic matrix support, which can easily be obtained using agricultural or cheap food wastes^45^. Also, this study was related to the investigation of the synergistic effect between ZnO NPs and EEP in terms of antimicrobial properties against prokaryotic cells of *E*. *coli*, *B*. *Subtilis*, and eukaryotic cells of *C*. *albicans* strains, effect which was not taken into consideration until know to our knowledge.

## Experimental

### Materials

Bacterial cellulose (BC) was obtained in Mass Transfer Laboratory of University Politehnica of Bucharest by employing a modified method of Hestrin - Schramm [MHS] culture medium with 2% wt. carbon source using wastes of forest fruits in a static culture at 28 °C for 7 days^46^. The BC pieces were microbiologically inactivated in an aqueous solution of NaOH 0,5 N at 90 °C for 1 hour and then rinsed repeatedly with deionized water until neutral pH. Raw propolis produced by *Apis mellifera carnica* bee specie was purchased from local beekeepers of Dolj County, Romania and used without further purification. Zinc acetate (Sigma-Aldrich), ammonia (25 wt.%) (Sigma-Aldrich), ethanol (96% vol.) (Redox) were used as such. *Escherichia Coli* (K12-MG1655)., *B*. *subtilis Spizizenii Nakamura* (ATCC 6633), *Candida albicans* (ATCC10231) were selected from the strain collection of Chemical and Biochemical Engineering Department of University Politehnica of Bucharest.

### Methods

#### Ultrasound-assisted synthesis of ZnO NPs

In a 50 mL round flask 0.07 g of zinc acetate and 20 mL of distilled water were added. Ammonia (25% wt.) was further added until a pH of 11 was reached. The reactions were kept for 2 min at temperature of 60 °C immersed in ultrasonic bath at two different frequencies of 40 kHz, respectively 100 kHz. The samples were encoded ZnO-40 US and ZnO-100 US. For comparison ZnO NPs were obtained after 2 min of continuous stirring by conventional method (without ultrasounds) in the absence of BC keeping the rest of the parameters, constant.

#### Ultrasound synthesis of BC-ZnO films

The purified BC was cut in small slices with dimensions of 4 × 3 cm. Thus, 22 g of BC (98 % wt. moisture) were stirred in a solution of zinc acetate (0.4987 g) previously dissolved in 143 mL of distilled water. Ammonia (25 % wt.) was added until pH = 11 was reached in order to obtain the ZnONPs on the surface of the BC structure. The reactions were performed considering the same frequencies as for blank ZnO (*Section 2*.*2*.*1*). The BC-ZnO films were dried until constant mass and further cut into discs with diameter of 6 mm.

#### Preparation of propolis extracts

Four different concentrations of propolis aliquots(3.5 % wt, 7 % wt., 11 % wt, and 15 % wt assigned as EEP1, EEP2, EEP3, respectively EEP4) were prepared using raw propolis and ethanol (96 % vol.) as solvent by continuous stirring for 24 hours. The clear yellowish-orange alcoholic extracts were recovered after centrifugation and used as such to modify the BC-ZnO films.

#### Preparation of BC-ZnO-propolis films

BC-ZnO discs were impregnated with propolis extracts in three sequential steps (deposition of 1 μL of EEP/step) in order to cover the whole surface of the BC-ZnO substrates.

### Characterization

A FEI Quanta Inspect F scanning electron microscope (SEM) coupled with an energy-dispersive X-ray (EDX) detector was used for the morphological investigation of the BC-ZnO composite materials; in order to achieve conductive surfaces, the samples were coated with a thin layer of gold (few nm) by DC sputtering.

The as-prepared composites were subjected to thermal analysis in the 20–1000 °C temperature range, in air, with the help of a Shimadzu DTG-60 equipment.

The formation of ZnO layer and organic propolis deposited on BC substrate were analyzed by XPS EnviroESCA^TM^ (SPECS^TM^ Surface NanoAnalysis GmbH) equipped with monochromatic Aluminum Kα excitation (hν = 1486.7 eV). The measurements were performed at 1mbar in inert atmosphere of Argon after fixing the samples with Carbon tape on standard plate. Sample deconvolution was carried out using C 1 s hybridization peak at 285.0 eV.

The antioxidant activity of the propolis samples was evaluated using DPPH (2,2-diphenyl-1-picrylhydrazyl) free radical neutralization assay according to^47^ with slight modifications (described in *Supplementary Information*).

Gas chromatography–mass spectrometry (GC–MS) analysis was performed on a Clarus 680 Perkin Elmer gas chromatograph equipped with a MS TurboMass-Perkin Elmer detector. The chromatographic column used for the analysis of propolis extracts was Elite 35 –MS (35% Diphenyl/65% Dimethyl polysiloxane, 30 m × 0.25 mm i.d. × 0.25 μm film thickness). Helium (99.999% purity), the carrier gas, was introduced at a flow rate of 1 mL/min. The propolis samples were analyzed by keeping a strict temperature regime of the chromatographic column: first at 100 °C for 2 min, then increased to 200 °C with 10 °C/min and further kept at 200 °C for 2 min. In the end, the temperature was raised with 5 °C/min to 280 °C and kept at this temperature for 9 min/sample. The samples (2 μL) were injected in split mode at 250 °C.

The antimicrobial tests were performed using the disc diffusion method. The discs used in antimicrobial experiments have controlled dimensions (diameter 6 mm ± 0.2, and thickness of 0.2 mm ± 0.05) and their sterilization was performed using UV lamp (UVGL-58, Multiband UV, UVP, U.S.A.) at 254 nm for 30 minutes. For bacterial strains Nutrient Agar was used (Carl Roth GmbH + Co.KG), while for yeast strain PDA (Potato-Dextrose-Agar - Carl Roth GmbH + Co.KG) was chose as culture media. The whole culture media were sterilized by autoclaving (Raypa), at 121 °C for 20 min. The plates were inoculated using cell depletion technique with 0.1 mL cell suspension with concentration 0.5 ± 0.1 OD (at 600 nm) followed by incubation at specific temperature (37 °C for all strains) for 72 h.

## Results and Discussions

### Morphological characterization

**Figure 1:**
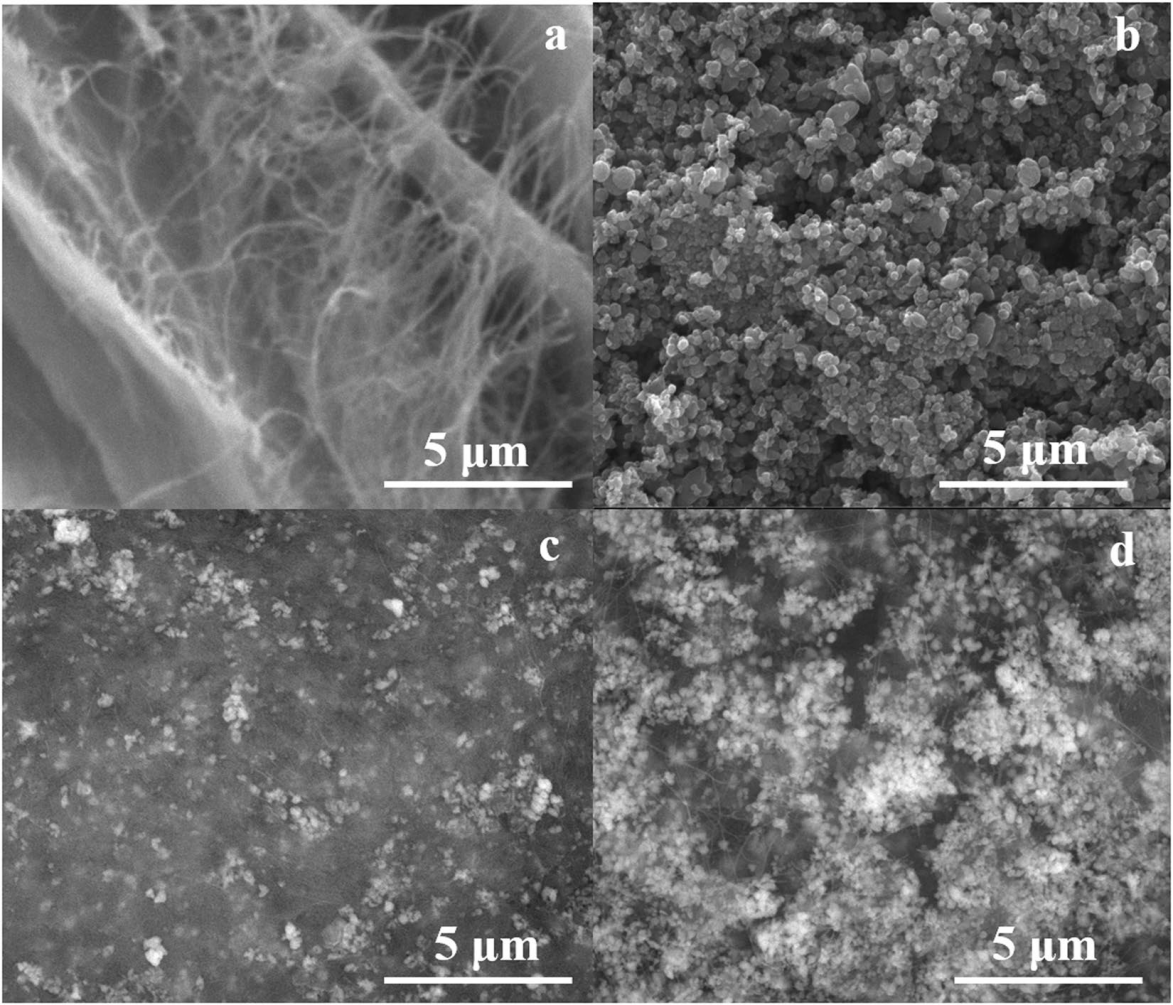
SEM micrographs of BC films (**a**), ZnO NPs (**b**), BC-ZnO-40US (**c**), and BC-ZnO-100US (**d**).

The first step in our research study was to investigate the morphology of unmodified BC films (Fig. 1a), respectively ZnO NPs obtained in the absence of BC and ultrasounds (Fig. 1b).

The BC films presented an irregular distribution of the cellulose fibers confirming the porous areas of the membranes according to literature data^48^.

Research data related to unconventional methods used for the synthesis of nanoparticles revealed that there is a strong connection between controlled sizes and antimicrobial activity^49^.

In our case, in the absence of ultrasounds the inorganic ZnO structures had different sizes and shapes (Fig. 1b), while in the case of the assisted-ultrasound synthesis method, at 40 kHz (BC-ZnO-40US), the inorganic nanoparticles appear uniformly distributed due to a reduced growth effect of the particles^50^ (Fig. 1c). By analysing few tens of quasi-spherical particles in the case of each sample, particle diameters in the range 70 ÷ 90 nm were determined for 40 and 100 kHz frequency respectively, most of the time being gathered as aggregates with sizes up to 1 μm. The presence of ultrasounds allows a better control of the obtaining process of relatively monodisperse inorganic nanoparticles^50^ and the application of ultrasounds determined the entrapment of ZnO NPs not only on the surface of the BC films, but also in the porous parts of the membranes forming agglomerated structures between the irregular fibers (Fig. 1c). The increase of frequency (100 kHz) during the deposition of the inorganic particles led to a higher amount of ZnO NPs deposited on the surface and larger agglomerates embedded deeper down in the BC structure for BC-ZnO-100US films (Fig. 1d).

### EDX analysis on BC-ZnO films

**Figure 2:**
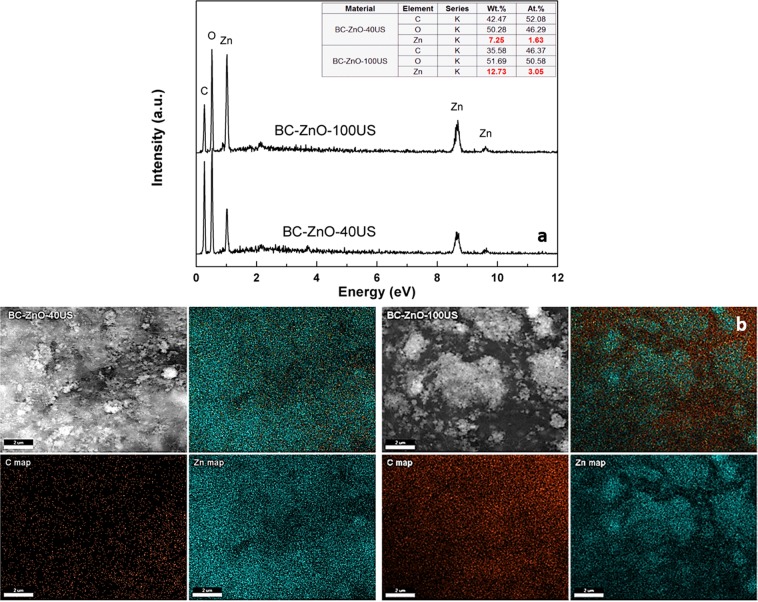
EDX analysis performed on both types of composite films obtained at 40 kHz, respectively 100 kHz: (**a**) spectra and elemental composition and (**b**) elemental mapping.

The formation of ZnO on the BC films was confirmed by EDX analysis (Fig. 2). As expected, the composite films obtained at 100 kHz registered almost a double amount of metallic element on their surface (12.73 wt.%), compared to those obtained at 40 kHz (7.25 wt.%) which are in agreement with the results attained by SEM analyses. The carbon signals belong to the BC natural polymer. Moreover, in order to demonstrate the homogeneous distribution of inorganic phase on the surface of BC substrate, compositional maps (Fig. 2b) were recorded for atoms of interest: C as representative for BC and Zn for ZnO, O being common for both of them. As it can be observed, in both cases, Zn concentration does not show significant gradients from one area to another, thus confirming an uniform distribution of the inorganic particles on BC surface.

### TGA analysis of BC-ZnO films

The BC-ZnO composite films were investigated in order to evaluate the thermal behavior of the hybrid materials and the ZnO NPs loading on the BC membrane.

**Figure 3:**
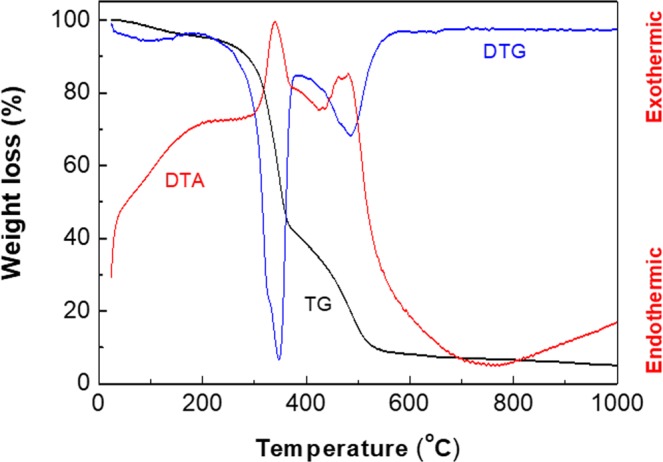
Thermal analysis of BC modified with ZnO.

In Fig. 3 the thermo-gravimetric (TG, DTG), respectively the differential thermal analysis only for BC-ZnO-100US is presented, since the thermal behaviour of our two samples obtained at different frequencies was similar. The first temperature range, from room temperature to 200 °C, are characterized by minor weight loss (5%) and can be explained by the endothermic removal of moisture adsorbed on the surface of the samples.

In the second stage, an accentuated weight loss occurs between 200 and 380 °C, which can be attributed to the exothermic decomposition of BC membrane (Fig. 3).

In this temperature range, the weight loss of the composites was 57 % for BC-ZnO-40US, respectively 62 % for BC-ZnO-100US. The final significant weight loss stage was registered between 380 °C and 580 °C for both samples and associated with an exothermic effect which ca be assigned either to a more crystalline form of BC having a better thermostability or to a mixing effect, the mineral phase on the surface of BC fibre enhancing their thermal properties. After complete removal of the gas generating components, the total amount of the inorganic particles remaining in the samples was around 5% wt.

### XPS analysis of the BC-ZnO-propolis composites

In order to obtain more informationson the chemistry of the pristine and modified BC, high-resolution spectra were performed.As expected, signals for carbon, nitrogen and oxigen were registered for all samples.

**Figure 4:**
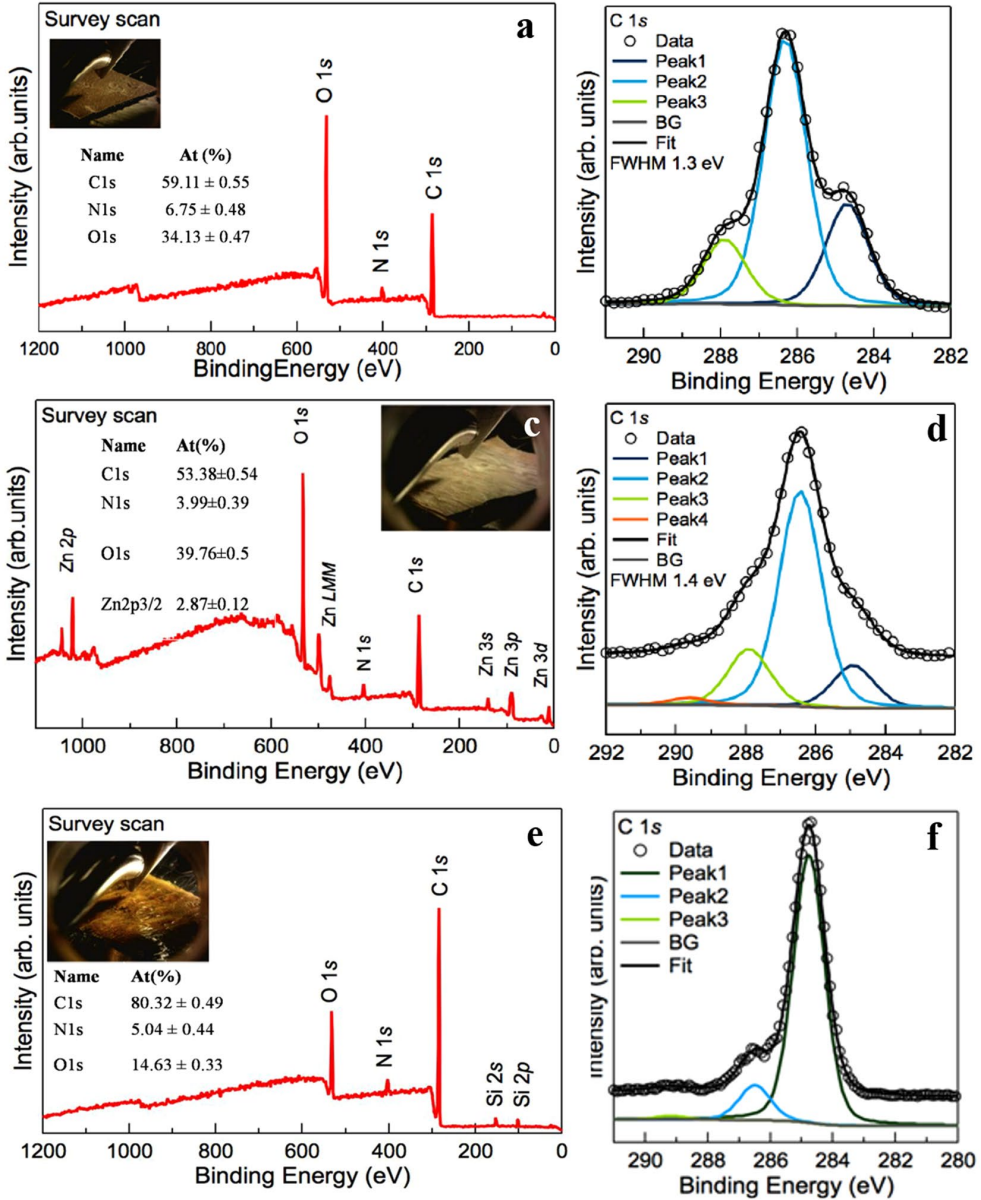
XPS survey over the surface of: (**a**) BC; (**c**) BC-ZnO, (**e**) BC-ZnO-propolis films, respectively deconvolution of C1s peak (**b**,**d**,**f**).

At the binding energy of 285.0 eV,C1s was assigned to un-oxidiezed carbon atom from C-H, C-C, respectively C=Cgroups. The signals registered at 286.5 eV and 288 eV were attributed to C-O single bond and C=O double bond. The composition of pristine BC (Fig. 4a) and BC-ZnO films (Fig. 4c) (Taokaew *et al*., 2015)^51^ is mostly formed by carbon (aproximatley 53÷60%), while in the case of BC-ZnO-propolis films (Fig. 4e) it reaches around 80%. In this study, our purpose was to use pristine BC without any supplimentary purifications (except the ones mentioned in *Section 2*.*1*). For this reason, N1s scans were also performed on all samples. The signals registered at 400 eV were assigned to free amine (H_2_N-C) and amide (O=C-N-C) groups which are coming from residual proteins of bacteria in the case of pristine BC (Fig. 4a) and from propolis organic components (Fig. 4e). For the samples modified with inorganic particles the XPS spectra for ZnO was registered at 1021 eV, thus confirming the presence of ZnO layer on the surface of the BC film (Fig. 4c). One can notice that the peaks from ZnO overlapp the peaks of Zn metal which was also confirmed by other literature studies(assigned as Zn2p3/2 spectrum) (Al-Gaashani *et al*., 2013^52^; Biesinger *et al*., 2010^53^). In the case of BC-ZnO-propolis composite materials XPS analysis revealed that the BC-ZnO films were completely covered with a layer of propolis and few disruptures appear in the material (silica signals coming from the standard substrate were registered) (Fig. 4e).

### Antioxidant properties of propolis

The antioxidant activity of ethanolic extracts of propolis was determined using the DPPH method (as described in *Supplementary information*). Samples containing raw propolis and EEP with lowest concentration of propolis (3.5% wt.) were evaluated. The results for radical scavenging activity (RSA) indicate that the solid sample of propolis and EEP (3.5% wt.) are slightly similar: 64.25% for raw propolis and 57.78% respectively. These results indicate a high antioxidant activity, sustained by the increased content of polyphenols and flavonoids^54,55^ also confirmed by GC-MS analysis (Table S1 *Supplementary information*).

### Antimicrobial results

The microorganisms selected in this study were both saprophytic and pathogenic, with a large spreading in human body and natural environment.*Candida albicans* is a pathogenic yeast and represents a common member of the human gut flora^56^. *Bacillus subtilis*, is a Gram-positive, catalase-positive bacterium, which forms spores; it is also found in soil and the gastrointestinal tract of ruminants^57^. *Escherichia coli* is a Gram-negativebacteria which can be found in contaminated water or food^58^ and human body. Its pathogenic effect has increased lately due to the abuse of antibiotics, which determined the increase of bacteria resistance^59^.

Thus, our purpose involved the use of ZnO and EEP as an alternative to commercial antibiotics in order to prevent the enhancement of the bacteria resistance.

The blank EEP solutions (deposited on filter paper) were studied in order to evaluate their antibacterial effect against pathogen Gram-negative bacteria and yeast, respectively on saprophyte Gram-positive bacteria. The results indicate a minimum inhibition concentration (MIC) smaller than 0.438 mg/mL for all EEP solutions tested (Figs. S1–S3 **- ***Supplementary information*).

In Table 1, the antimicrobial activity is presented for all samples to highlight a possible synergistic effect between all the components of the hybrid materials. In the first step, antimicrobial studies were performed for EEP deposited on pure BC films while using ethanol on BC films as control sample. As it can be observed, in the case of EEP the antimicrobial effect decreases in the order *B*. *subtilis* > *C*. *albicans* > *E*. *coli*. In the case of ZnO particles, regardless of the ultrasound frequency used to obtain BC-ZnO composites, the antimicrobial effect was noticed only in the case of *B*. *subtilis*. At reduced frequency, the two components (EEP and ZnO) induced a synergistic effect upon Gram positive bacteria (*B*. *subtilis*) and yeast (*C*. *albicans*) compared to Gram negative (*E*. *coli*) bacteria which are not sensitive to this synergistic effect. The increase of the frequency level for the synthesis of ZnO particles in the presence of BC substrate did not improved the antimicrobial activity considering the synergistic effect except for C. albicans at higher concentrations of EEP (EEP3, respectively EEP4).

**Table 1:** Antimicrobial activity of composite films against *E*. *coli*, *B*. *subtilis*, *C*. *albicans*.

Composite film code with EEP concentration	*E*. *coli*		*B*. *subtilis*		*C*. *albicans*	
	IZ, mm	MIC, mg/mL	IZ, mm	MIC, mg/mL	IZ, mm	MIC, mg/mL
BC -ETOH (control)	2 ± 0.05	1.89	0 (+)	>1.89	2 ± 0.07	1.89
BC-EEP1 (control) (S1^*^)	3 ± 0.1	1.3	8 ± 0.25	<0.44	3 ± 0.15	1.3
BC-EEP2 (control) (S2^*^)	1.5 ± 0.05	>0.8	9 ± 0.35	<0.44	4 ± 0.25	0.44
BC- EEP3 (control) (S3^*^)	0 (+)	>1.89	8 ± 0.22	<0.44	4 ± 0.25	0.44
BC- EEP4 (control) (S4^*^)	5 ± 0.25	0.44	12 ± 0.05	< 0.44	7 ± 0.1	<0.44
BC-ZnO-40 (control)	0 (+)	>1.89	4 ± 0.13	0.44	0 (−)	>1.89
BC-ZnO-40-EEP1 (F1S1^*^)	0 (+)	>1.89	5 ± 0.15	0.44	0 (+)	>1.89
BC-ZnO-40-EEP2 (F1S2^*^)	0 (+)	>1.89	8 ± 0.25	<0.44	1 ± 0.05	>0.8
BC-ZnO-40-EEP3 (F1S3^*^)	0 (+)	>1.89	4 ± 0.05	0.44	3 ± 0.35	1.3
BC-ZnO-40-EEP4 (F1S4^*^)	0 (+)	>1.89	4 ± 0.35	0.44	2 ± 0.05	1.89
BC-ZnO-100 (control)	0 (+)	>1.89	4 ± 0.15	0.44	0 (+)	>1.89
BC-ZnO-100-EEP1 (F2S1^*^)	0 (+)	>1.89	4 ± 0.25	0.44	0 (+)	>1.89
BC-ZnO-100-EEP2 (F2S2^*^)	0 (+)	>1.89	7 ± 0.15	<0.44	0 (+)	>1.89
BC-ZnO-100-EEP3 (F2S3^*^)	0 (+)	>1.89	3 ± 0.25	0.44	5 ± 0.15	1.3
BC-ZnO-100-EEP4 (F2S4^*^)	0 (+)	>1.89	2 ± 0.15	0.44	4 ± 0.25	1.89

^*^Assigned codes for BC-ZnOfilms impregnated with EEP solutions from Supplementary information.

Due to a larger inhibition zone (IZ) of EEP2 compared to EEP3, respectively EEP4, the diffusion of EEP with lower concentration from BC-ZnO-propolis films is higher. The diffusion phenomenon is evidenced by yellowish area surrounding the BC-ZnO-propolis films in Fig. S4, respectively Fig. S7 for both types of composite films BC-ZnO-40US (assigned as **F1** in *Supplementary information*), and BC-ZnO-100US (assigned as **F2** in *Supplementary information*). In the same time, the diffusion is also confirmed by mass inhibition phenomenon proved by clear areas corresponding to IZ (Figs. S4 and S7). In this case, the MIC value for *B*. *subtilis* was found 0.438 mg/mL (Table 1).

The BC-ZnO composite films modified with different concentrations of EEP did not show inhibition against the *E*. *coli* strain, the MIC for these probes being higher than 1.89 mg/mL corresponding to EEP4. Thus, in this case, for Gram-negative bacteria our films require a higher concentration of propolis (Table 1). Furthermore, the synergistic effect of BC modified with both EEP and ZnO did not influenced the growth of *E*. *coli* (Table 1**)** regardless of the EEP concentration or the frequency applied for inorganic particles synthesis (Figs. S5, respectively S8 **–** *Supplementary information*). This behavior could be attributed to the thick and continuous organic pellicle of propolis extracts that inhibit the diffusion of ZnO.

For the eukaryotic cells, *C*. *albicans*, the ZnO nanoparticles alone did not manifest antimicrobial effect, but in the interaction with EEP, at higher concentrations, EEP3 forBC-ZnO-40US, respectively EEP4 for BC-ZnO-100US, the antimicrobial effect is present with a significant IZ (Table 1). In the case of BC-ZnO-40US film the MIC value was 0.8 mg/mL, respectively 1.3 mg/mL for BC-ZnO-100US film. These results indicated that the film BC-ZnO-100US has a higher antimicrobial activity against *C*. *albicans* (as shown in Fig. S6 compared to Fig. S9).

The antimicrobial activity of propolis is determined by the presence of phenolic components such as flavonoids^60^. The antimicrobial effect of propolis is based mostly on the cell membranes degradation that led to a loss of potassium ions and finally to the cell autolysis^55,61^. Quercetin is a flavonoid often found in all types of propolis that determines the increasing of the membrane’s permeability causing the cell material loss^62^. In these circumstances bacterial motility is reduced to zero, and also membrane transport ability and capacity to synthesis adenosine triphosphate (ATP) is lost.

Our study revealed that propolis had a stronger influence upon the Gram-positive bacteria compared to Gram-negative and eukaryote cells. In general, natural extracts have normally a higher activity against Gram-positive bacteria than Gram-negative bacteria^59^, thus confirming our results. Gram-negative bacteria and eukaryote cells are more resistant than the Gram-positive due to a more complex chemical structure of the surrounding layers. The cell wall of Gram-negative bacteria contains polysaccharide, which has an important role in the antigenicity, toxicity and pathogenicity of the microorganisms. Furthermore, these bacteria also possess a higher lipid amount compared to Gram-positive bacteria^63^. Thus, the endotoxin (a lipopolysaccharide complex) is responsible for the diminished antimicrobial effect of the propolis or ZnO NPs or both, acting as a self-preserving mechanism of the cell.

Phenolic acids such as ferulic (compounds found in our samples – Table S1 **-** *Supplementary information*) and gallic acids disturb both the cell membranes of Gram-positive and Gram-negative bacteria. The effect consists in the changes of cell surface hydrophobicity and charging of the cell wall, which also is responsible for the leakage of cytoplasmic content^64^. The caffeic acid derivative has a similar effect on *Candida* cytoplasmatic membrane^65^. Another possible effect on the *C*. *albicans* cell wall can be attributed to caffeic acid derivatives that interact with 1,3-β-glucan synthase enzyme^66^.

The results obtained in this study showed that the antimicrobial activity of propolisis related to the total phenol content of the EEP extracts as shown in Table S1 (*Supplementary information)*.

Nevertheless, the effectiveness of bee’s products depends on differences regarding the chemical composition of the product, bee species and the pedoclimatic conditions of the region^63^. Thus, our study could be considered as an alternative to develop composite materials with selective antimicrobial activity against Gram-positive bacteria and yeast due to a synergistic effect between ZnO particles and EEP.

## Conclusions

In conclusion, we have obtained eco-friendly biodegradable BC films modified with ZnO NPs by ultrasound-assisted synthesis method impregnated with EEP solutions for possible future applications in food packaging with antimicrobial properties.

The BC-ZnO-propolis films have been characterized to evidence the deposition of ZnO, respectively EEP and their morphologic aspect by SEM, TGA, EDX, XPS. The composition of raw propolis and EEP solutions were determined by GC-MS, while the antioxidant activity was evaluated by DPPH method.

For the antimicrobial tests the BC-ZnO-propolis films were put onto culture media inoculated with *E*. *coli*, *B*. *subtilis*, and *C*. *albicans*. *E*. *coli* has proved to be highly resistant to the synergistic effect of both ZnO NPs and EEP concentration in all cases.

The synergistic effect between ZnO NPs and EEP acts upon *B*. *subtilis* at lower concentrations of EEP (EEP2) correlated with 40 kHz frequency applied for the synthesis of ZnO NPs, while in the case of *C*. *albicans*, the synergistic effect was more pronounced at enhanced concentration of EEP (EEP3) and higher ultrasound frequency (100 kHz).

Unexpectedly, the synergistic effect was diminished at the highest concentration of EEP for both BC-ZnO-40-EEP4 and BC-ZnO-100-EEP4 due to the formation of a compact pellicle of propolis organic compounds that acted like a barrier in the diffusion process of ZnO NPs.

## Supplementary information

Supplementary Information
