# Supplementary material for: Bacterial cellulose films with ZnO nanoparticles and propolis extracts: Synergistic antimicrobial effect
Source: Sci Rep. 2019 Nov 27;9:17687. doi: 10.1038/s41598-019-54118-w (PMC6881355; doi:10.1038/s41598-019-54118-w)
Supplement: Supplementary file 1 — Supplementary Information [file 41598_2019_54118_MOESM1_ESM.docx]

**Bacterial cellulose films with ZnO nanoparticles and propolis extracts:**

**Synergistic antimicrobial effect**

*(supplementary information)*

Alexandra Mocanu^1^, Gabriela Isopencu^1*^, Cristina Busuioc^1^, Oana-Maria Popa^2^,

Paul Dietrich^3^, Liana-Socaciu Siebert^3^

*^1^University POLITEHNICA of Bucharest, Faculty of Applied Chemistry and Materials Science,*

*Gh. Polizu Street 1-7, Bucharest, Romania, postal code 011061*

*^2^Research Center for Instrumental Analysis – SCIENT,*

*Petre Ispirescu Street 1, Tâncăbeşti, Ilfov, Romania, postal code 077167*

*^3^SPECS Surface Nano Analysis GmbH, Voltastrasse 5, 13355 Berlin, Germany*

*e-mail corresponding author:gabriela.isopencu@gmail.com; g_isopencu@chim.upb.ro*

**Antioxidant properties of propolis**

Brieﬂy, propolis extract solution was mixed with 1 mM ethanolic solution of DPPH. The mixture was stirred using a vortex-BioCote and incubated in the dark at ambient temperature for 30 min. When the DPPH solution was mixed with the sample mixture acting as a hydrogen atom donor, a stable nonradical form of DPPH is obtained with simultaneous change of the violet color to pale yellow. The absorbance was then measured at 517 nm. The percentage of DPPH free radical quenching (RSA) activity was determined using the following equation:

$${RSA=DPPH}_{scavenging effect}=\frac{{Abs}_{DPPH}-{Abs}_{extract}}{{Abs}_{DPPH}}$$

where Abs_DPPH_ is the absorbance value at 517 nm of the ethanolic solution of DPPH, and Abs_extract_ is the absorbance value at 517 nm for the sample extracts. Each sample was assayed at least ﬁve times.

***GC-MS analysis of propolis***

**Table S1.** *The composition of propolis extract determined by GC-MS analysis*

| RT | Compounds |
| --- | --- |
| ***Flavonoids*** | |
| 51 | Methyl Chrysin / Tectochrysin- |
| 37 | Naringeninic Acid |
| ***Alifatic Acids*** | |
| 23.7 | Eicosanoic Acid |
| 21.21 | Octadecadienoic Acid |
| 20.99 | 2-Propenoic Acid, 3-(4-Hydroxy-3-Methoxyphenyl) |
| 20.91 | 2-Propenoic Acid, 3-(4-Hydroxy-3-Methoxyphenyl) / Ferulic Acid |
| 19.91 | 2-Propenoic Acid, 3-(4-Hydroxyphenyl) |
| 13.81 | Hexadecanoic Acid / Palmitic Acid |
| 11.44 | Tetradecanoic Acid / Myristic Acid |
| 9.48 | Glutaric Acid / N-Pyrotartaric Acid |
| 6.84 | Decanoic Acid / Capric Acid / Caprinic Acid |
| 2.27 | Tiglic Acid / Cevadic Acid/ Crotonic Acid, 2-Methyl-, (E)- |
| ***Aromatic Acids*** | |
| 23.29 | Cinnamyl Cinnamate / Cinnamic Acid, |
| 20.44 | Ferulic Acid |
| 18.7 | P-Coumaric Acid, Trans(3-(4-Hydroxyphenyl)-1-Propenoic Acid)/ Naringeninic Acid |
| 14.61 | 2-Propenoic Acid, 3-(4-Hydroxy-3-Methoxyphenyl)-, Methyl Ester / Methyl Ferulate / Ferulic Acid Methylester |
| 9.25 | Trans-Caffeic Acid |
| 5.26 | Benzoic Acid |
| 5.19 | Dracylic Acid |
| 5.19 | Phenylformic Acid |
| ***Esters*** | |
| 25.30 | N-Cbz-Glycyl-L-Tyrosine Benzyl Ester |
| 23.29 | Cinnamyl Ester |
| 21.21 | Methyl Ester, |
| 19.91 | 4-Coumaric Acid Methyl Ester/ Acrylic Acid, Sodium Salt Ester |
| 12.98 | Phloroglucinol Ester |
| 12.98 | Phloroglucinol Ester |
| 9.48 | Di(2-Nitrobenzyl) Ester / Pentanedioic Acid Ester |
| 7.74 | Diacetate / Glycerol 1,2-Diacetate / 2,3-Diacetin |
| 6.62 | Monoacetate / Acetin / Glyceryl Acetate |
| 6.52 | Diacetate / Glycerol Diacetate / 1,2-Diacetin/Glyceryl Diacetate |
| 5.26 | Ethyl Ester |
| 4.70 | Monoacetate/ Monoacetin/ Acetin / Glyceryl Acetate |
| ***Alcohols, Terpene, Quinone*** | |
| 11.84 | Phloroglucinol / Floroglucinol/ Benzene |
| 10.84 | Flamenol / 5-Methoxyresorcinol / Phloroglucinol Monomethyl Ether |
| 10.16 | Guaiol / Champacol / Guaiac Alcohol |
| 9.86 | Phenol, 2-Methoxy-4-Propyl/ P-Propylguaiacol/ Cerulignol |
| 7.52 | 2-Propen-1-Ol, 3-Phenyl / Alpha-(2-Phenylvinyl)-3-Pyridinemethanol Hydrochloride |
| 7.41 | 2-Methoxy-4-Vinylphenol / 4-Vinylguaiacol |
| 7.74 | 1,2,3-Propanetriol, Diacetate |
| 6.62 | 1,2,3-Propanetriol, Monoacetate |
| 6.52 | 1,2,3-Propanetriol, Diacetate |
| 4.83 | Benzeneethanol / Benzyl Carbinol / Phenethyl Alcohol |
| 4.70 | 1,2,3-Propanetriol, Monoacetate |
| 3.85 | Benzyl Alcohol/ Benzenemethanol / Phenylmethanol / Alpha-Toluenol |
| ***Others*** | |
| 26.83 | 4h-1-Benzopyran-4-One, 5-Hydroxy-7-Methoxy-2-Phenyl / Methyl Chrysin / Tectochrysin- |
| 25.88 | 4h-1-Benzopyran-4-One, 2,3-Dihydro-5,7-Dihydroxy-2-Phenyl-, (S) Pinocembrin / (S)-5,7-Dihydroxyflavanone |
| 25.19 | 4h-1-Benzopyran-4-One, 2,3-Dihydro-5,7-Dihydroxy-2-Phenyl-, (S) / Pinocembrin / (S)-5,7-Dihydroxyflavanone |
| 24.67 | 2',4'-Dimethoxy-3'-Methylpropiophenone / 2',4'-Dimethoxy-3'-Methylpropiophenone Ester |
| 24.01 | Flemichapparin |
| 20.99 | 9-Octadecenamide / Elaidamide / Sleepamide |
| 20.09 | Oxirane, [(Dodecyloxy)Methyl]- / Lauryl Glycidyl Ether / Dodecylglycidyl Ether |
| 18.13 | 9-Octadecenamide / Elaidamide/ Oleic Acid Amide |
| 18.13 | 9-Octadecenamide / Elaidamide/ Oleic Acid Amide |
| 17.12 | Octadecanal / Stearyl Aldehyde |
| 15.73 | Octadecanal / Stearyl Aldehyde |
| 15.51 | 2-Nonadecanone Methyl Heptadecyl Ketone |
| 14.40 | Octadecanal / Stearyl Aldehyde |
| 13.52 | Benzyl Benzoate / Ascabin / Scabitox/ Peruscabin |
| 9.15 | Vanillin/ Benzaldehyde, 4-Hydroxy-3-Methoxy / Lioxin / Zimco |
| 8.19 | 3-Buten-2-One, 4-Phenyl 4-Phenyl-1-Piperidino-4-(2-Thienyl)-3-Buten-2-One |
| 7.82 | 1-Phenyl-2-Hexanone / 1-Phenylhexan-2-One / Benzyl N-Butyl Ketone |
| 6.29 | 1-Phenyl-2-Butanone / Benzyl Ethyl Ketone |
| 5.09 | 2-Propanone, 1-Phenyl/ Benzyl Methyl Ketone / Phenylacetone |
| 4.29 | Ethanone, 1-Phenyl / Acetophenone |
| 3.23 | Propane, 2-Fluoro-2-Methyl / Tert-Butyl Fluoride |
| 3.14 | Benzaldehyde / Benzoic Aldehyde / Artificial Almond Oil |

***Antimicrobial analysis***


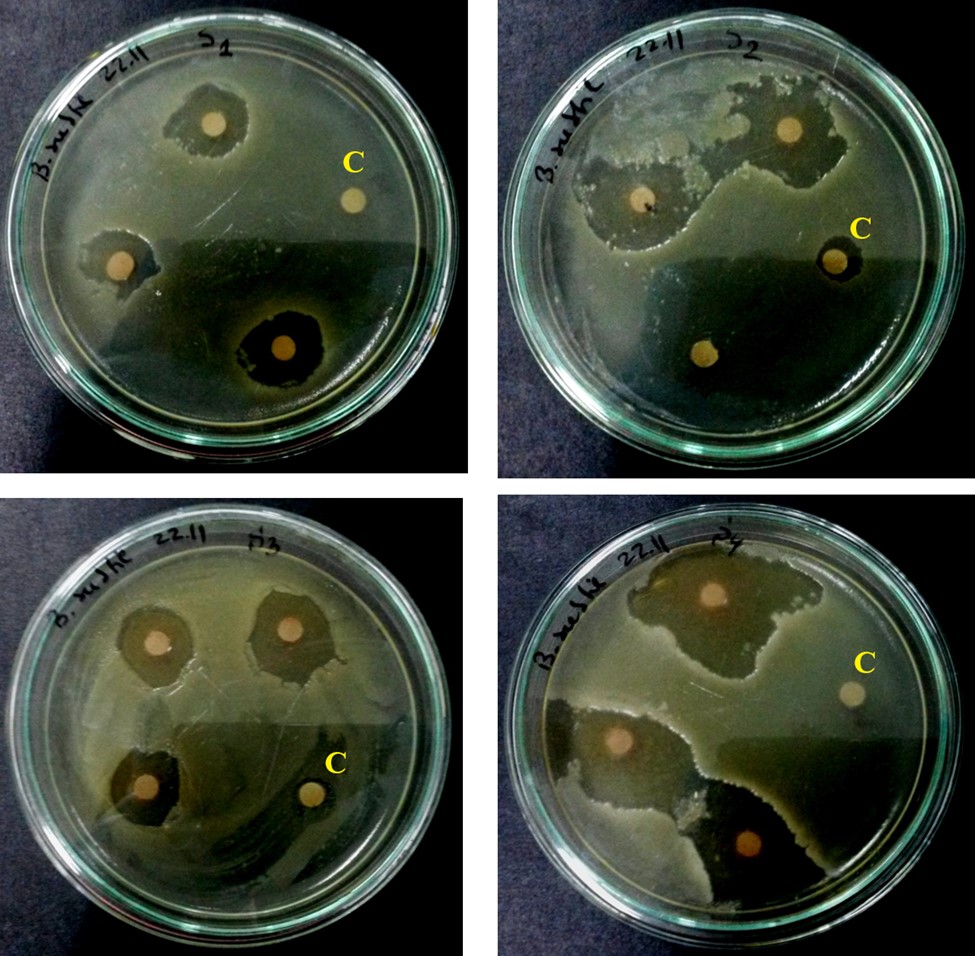


**Figure S1**. Antimicrobial activity of EEP with different concentration (S1-4) against *B. subtilis*, gram positive bacteria (three samples - yellow and a control sample – white)


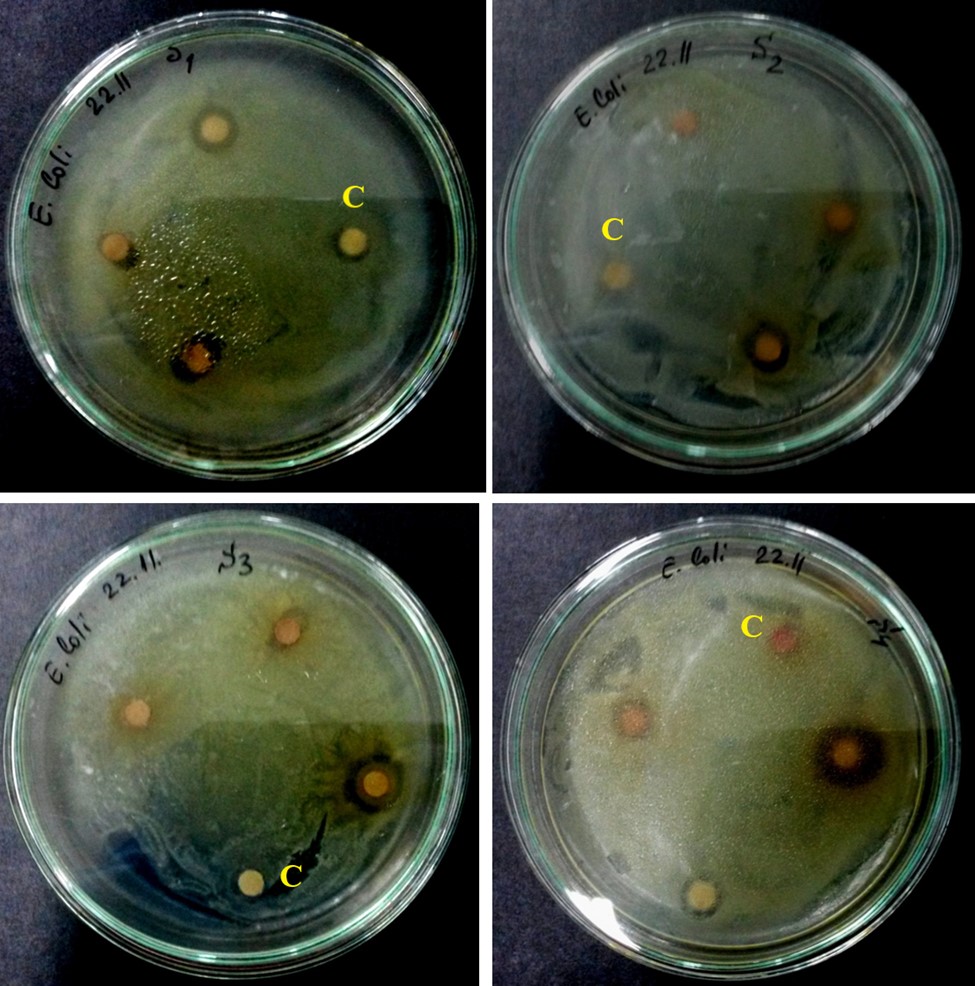


**Figure S2.** Antimicrobial activity of EEP with different concentration (S1-4) against *E. coli*, gram negative bacteria (three samples - yellow and a control sample – white)


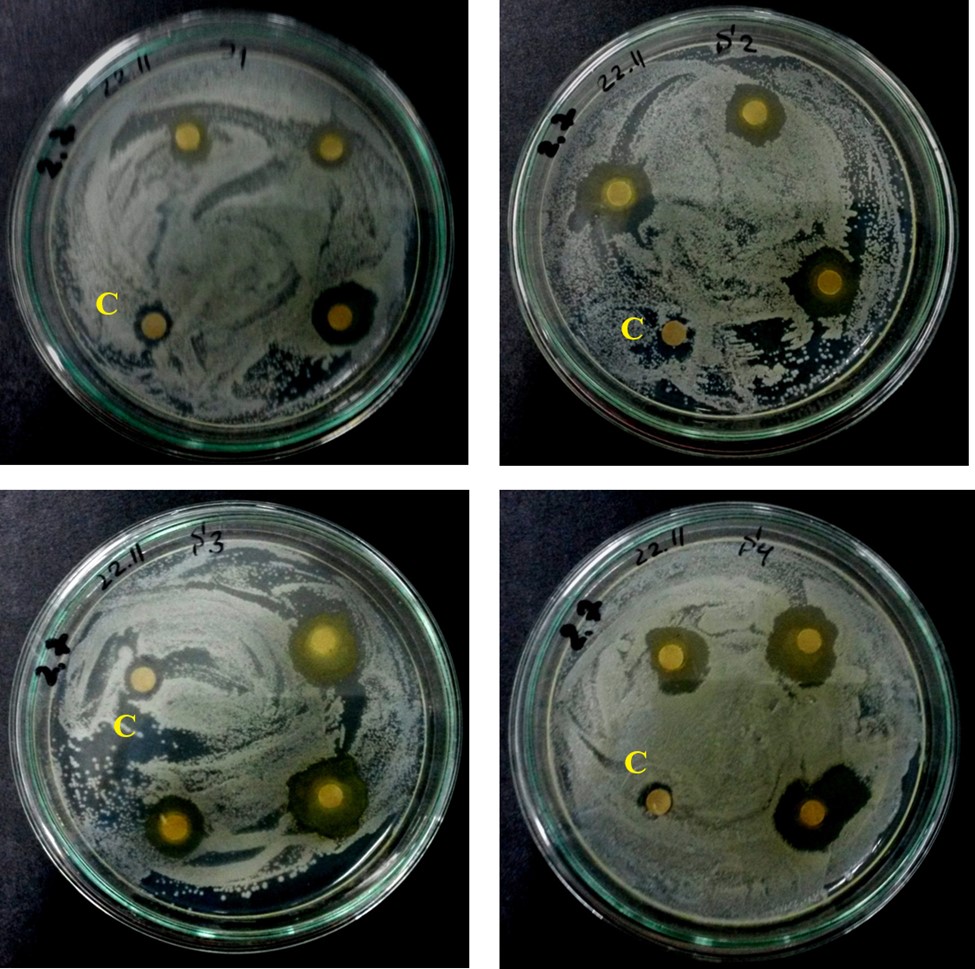


**Figure S3**. Antimicrobial activity of EEP with different concentration (S1-4) against *C. albicans*, yeast (three samples - yellow and a control sample – white)


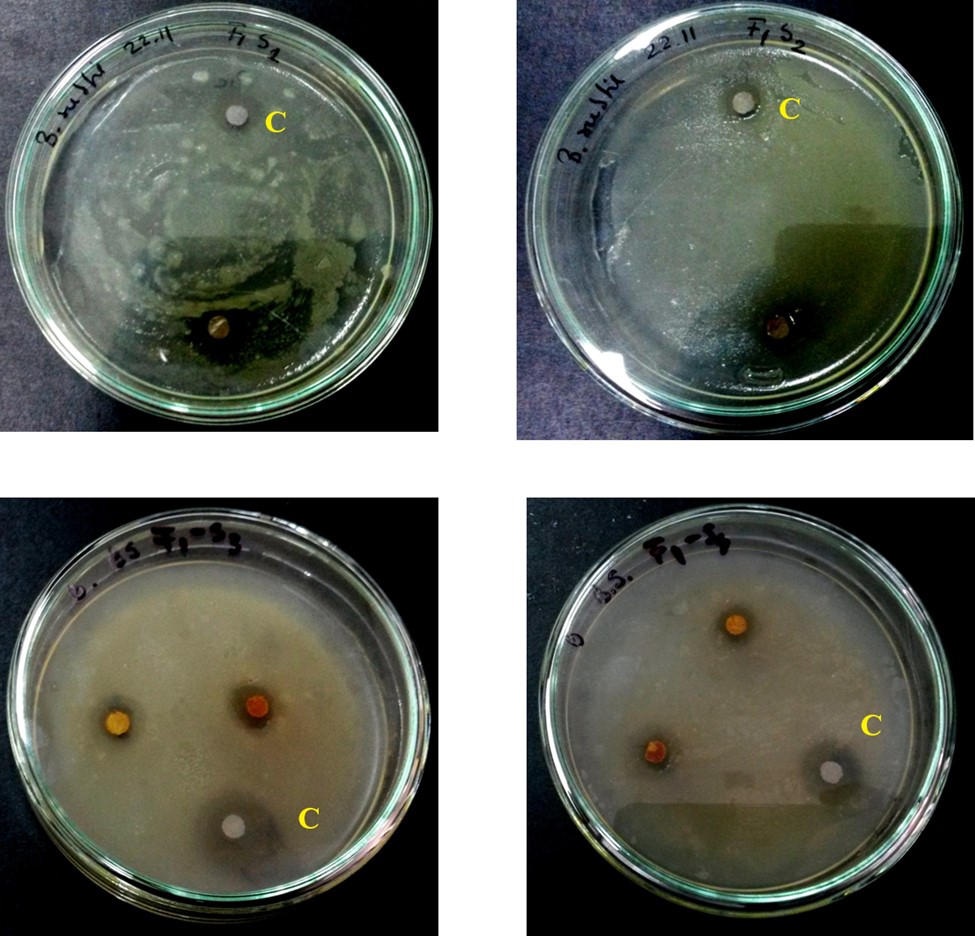


**Figure S4**. Antimicrobial activity of EEP with different concentrations (**S1÷S4**) on

BC-ZnO-40US (**F1**) composite film against *B. subtilis*

(1 or 2 samples – yellow/brown and a control sample (C) – white)


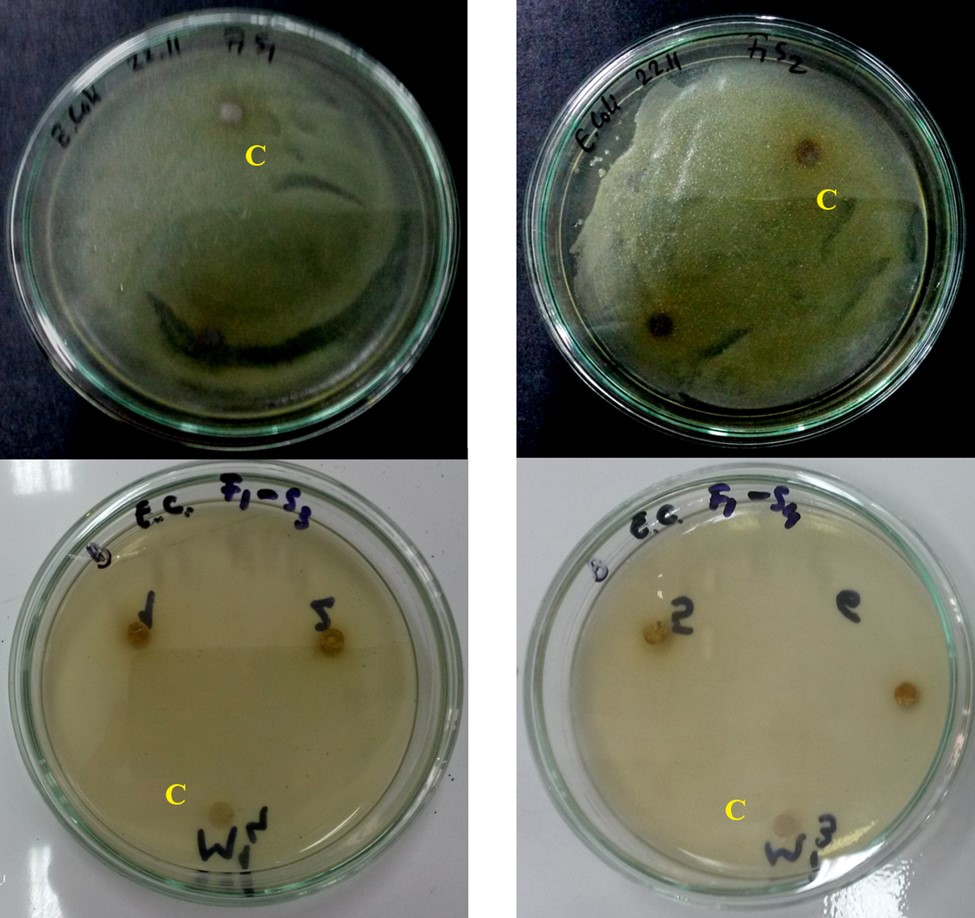


**Figure S5.** Antimicrobial activity of EEP with different concentrations (**S1÷S4**) on

BC-ZnO-40US (**F1**) composite film against *E. coli*

(1 or 2 samples – yellow/brown and a control sample (C) – white)


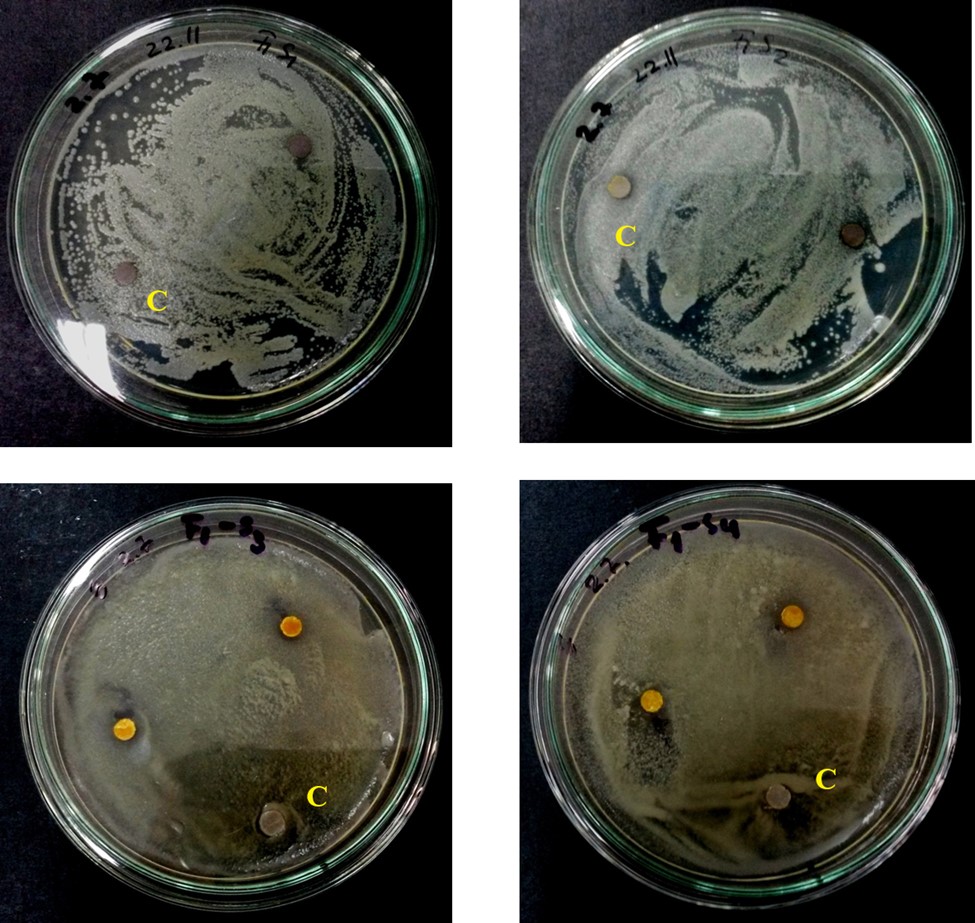


**Figure S6.** Antimicrobial activity of EEP with different concentrations (**S1÷S4**) on

BC-ZnO-40US (**F1**) composite film against *C. albicans*

(1 or 2 samples – yellow/brown and a control sample (C) – white)


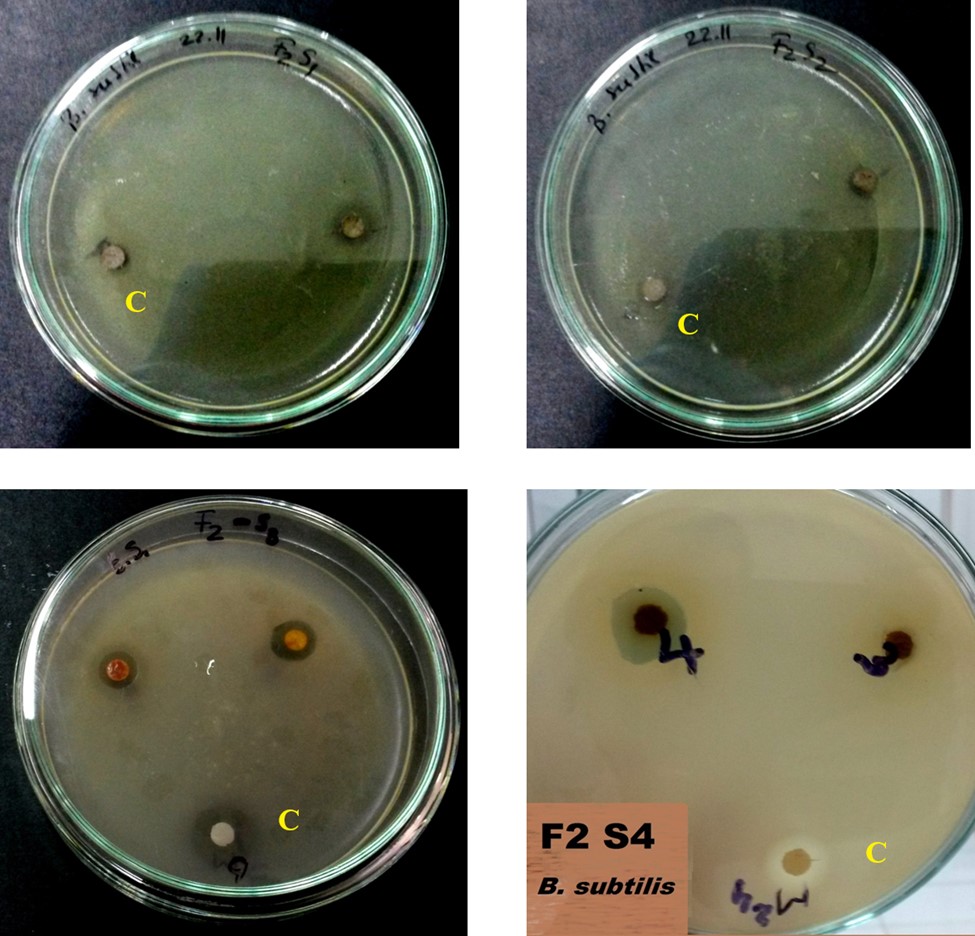


**Figure S7.** Antimicrobial activity of EEP with different concentrations (**S1÷S4**) on

BC-ZnO-100US (**F2**) composite film against *B. subtilis*

(1 or 2 samples – yellow/brown and a control sample (C) – white)


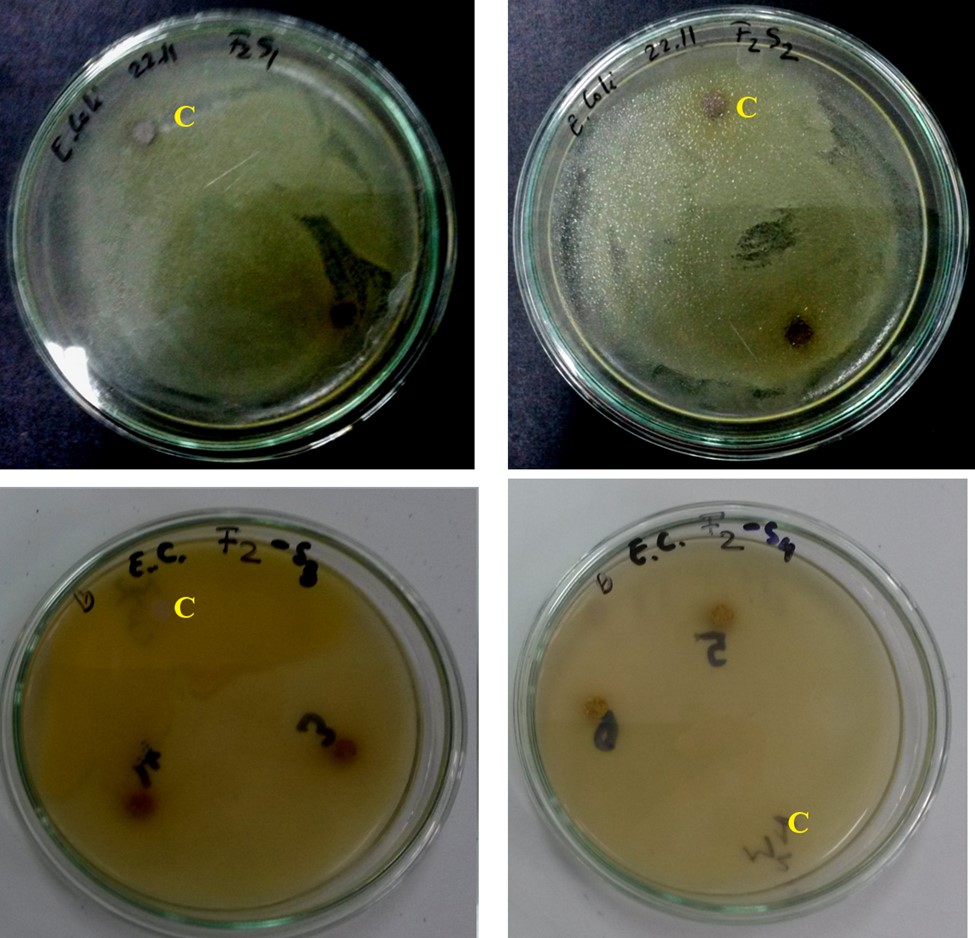


**Figure S8.** Antimicrobial activity of EEP with different concentrations (**S1÷S4**) on

BC-ZnO-100US (**F2**) composite film against *E. coli*

(1 or 2 samples – yellow/brown and a control sample (C) – white)


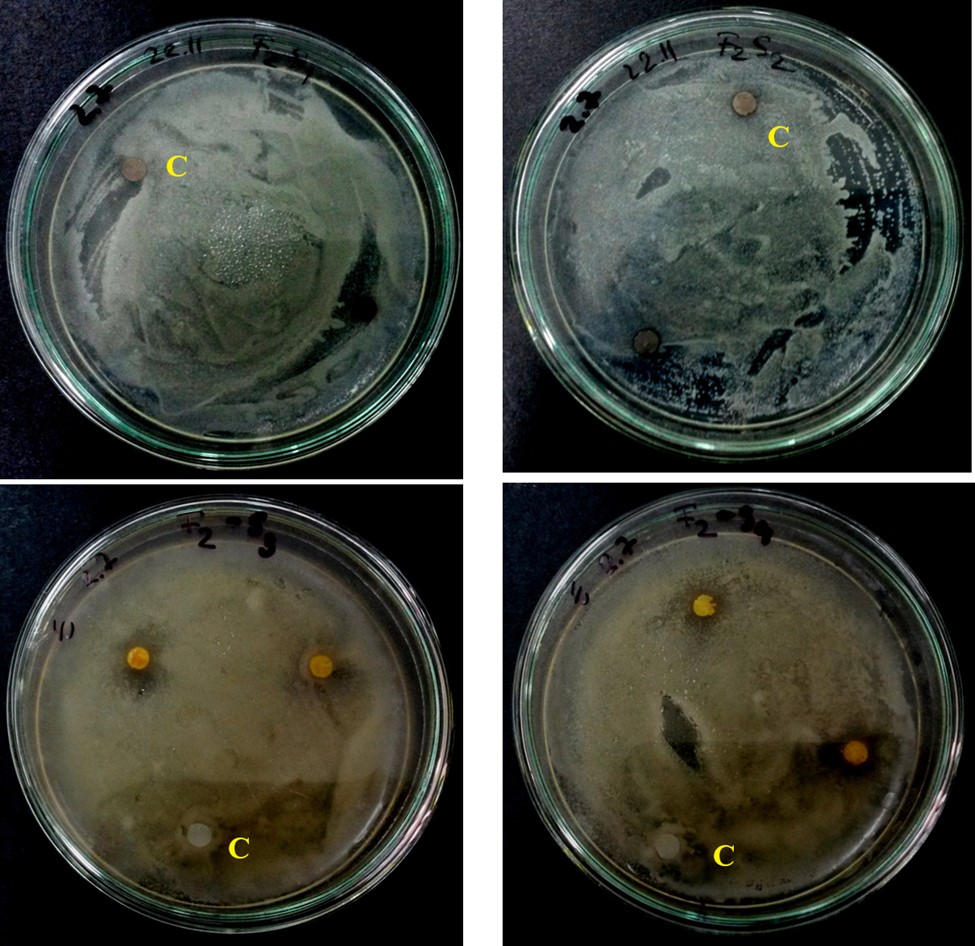


**Figure S9.** Antimicrobial activity of EEP with different concentrations (**S1÷S4**) on

BC-ZnO-100US (**F2**) composite film against *C. albicans*

(1 or 2 samples – yellow/brown and a control sample (C) – white)
